# Suppression of RNA interference increases alphavirus replication and virus-associated mortality in *Aedes aegypti *mosquitoes

**DOI:** 10.1186/1471-2180-9-49

**Published:** 2009-03-05

**Authors:** Chris M Cirimotich, Jaclyn C Scott, Aaron T Phillips, Brian J Geiss, Ken E Olson

**Affiliations:** 1 Arthropod-Borne and Infectious Diseases Laboratory, Department of Microbiology, Immunology and Pathology, Colorado State University, Fort Collins, CO 80523 USA; 2 Department of Biochemistry and Molecular Biology, Colorado State University, Fort Collins, CO 80523 USA; 3 Current address : Department of Molecular Microbiology and Immunology, Bloomberg School of Public Health, Johns Hopkins University, Baltimore, MD 21205 USA

## Abstract

**Background:**

Arthropod-borne viruses (arboviruses) can persistently infect and cause limited damage to mosquito vectors. RNA interference (RNAi) is a mosquito antiviral response important in restricting RNA virus replication and has been shown to be active against some arboviruses. The goal of this study was to use a recombinant Sindbis virus (SINV; family *Togaviridae*; genus *Alphavirus*) that expresses B2 protein of Flock House virus (FHV; family *Nodaviridae*; genus *Alphanodavirus*), a protein that inhibits RNAi, to determine the effects of linking arbovirus infection with RNAi inhibition.

**Results:**

B2 protein expression from SINV (TE/3'2J) inhibited the accumulation of non-specific small RNAs in *Aedes aegypti *mosquito cell culture and virus-specific small RNAs both in infected cell culture and *Ae. aegypti *mosquitoes. More viral genomic and subgenomic RNA accumulated in cells and mosquitoes infected with TE/3'2J virus expressing B2 (TE/3'2J/B2) compared to TE/3'2J and TE/3'2J virus expressing GFP. TE/3'2J/B2 exhibited increased infection rates, dissemination rates, and infectious virus titers in mosquitoes following oral bloodmeal. Following infectious oral bloodmeal, significantly more mosquitoes died when TE/3'2J/B2 was ingested. The virus was 100% lethal following intrathoracic inoculation of multiple mosquito species and lethality was dose-dependent in *Ae. aegypti*.

**Conclusion:**

We show that RNAi is active in *Ae. aegypti *cell culture and that B2 protein inhibits RNAi in mosquito cells when expressed by a recombinant SINV. Also, SINV more efficiently replicates in mosquito cells when RNAi is inhibited. Finally, TE/3'2J/B2 kills mosquitoes in a dose-dependent manner independent of infection route and mosquito species.

## Background

Arthropod-borne viruses (arboviruses) such as Sindbis and Chikungunya viruses are transmitted to humans through the bite of an infected mosquito. The viruses exhibit significant morbidity and mortality in the vertebrate host. However, virus persists in the mosquito vector with minimal associated pathology. Examples of arbovirus-induced cytopathology during infection have been described with laboratory-infected mosquitoes, but little is known about the interplay between virus and vector that allows for sustainable arbovirus infection in the environment [1-5]. The persistent nature of arbovirus infection of a vector suggests a commensal rather than parasitic relationship. A factor of particular interest in this relationship is the interaction of viral replication and the mosquito RNA interference (RNAi) response to infection.

RNAi is a highly conserved molecular pathway triggered by the presence of intracytoplasmic double-stranded RNA (dsRNA) that results in the cleavage of RNA molecules with sequence homologous to the dsRNA. In insects, RNAi is a major antiviral pathway that modulates arbovirus infection. Keene et al (2004) and Campbell et al (2008) used dsRNA injection to show that transient knockdown of key RNAi components increases viral loads in individual mosquitoes. Titers of O'nyong-nyong virus (ONNV) in *Anopheles gambiae *and Sindbis virus in *Aedes aegypti *were higher if Argonaute-2 or Dicer-2 expression was silenced [6,7]. These studies show that RNAi restricts replication of an arbovirus in the mosquito.

During replication of the alphavirus genome, positive- and negative-sense RNAs form dsRNA intermediates that could be recognized and cleaved by Dicer-2. Alternatively, secondary structure of the positive-sense RNA genome may be targeted by the RNAi machinery, as was shown in plants infected with positive-sense, ssRNA viruses [8,9]. SINV-specific siRNAs of both polarities have been detected in infected mosquitoes with increased sense siRNAs being observed [6,10], suggesting secondary structure is the primary, but not only, molecular RNAi trigger. Thus SINV replication appears to be targeted by the RNAi response in mosquitoes. To date there have been no reports of SINV-encoded proteins that interfere with the RNAi response, although certain strains of SINV appear to be more or less refractory to RNAi in mosquito cells [6]. However, other insect viruses are known to contain RNAi suppressors that aid in their replication via suppression of the RNAi response. The B2 protein from the insect-pathogenic FHV is a potent viral suppressor of RNA silencing (VSR) that binds to dsRNA as a dimer in a sequence-independent manner and can bind a range of dsRNA sizes [11,12]. The generic and promiscuous nature of dsRNA binding by B2, evidenced by its ability to inhibit RNAi in plants, nematodes, and insects, makes it an excellent candidate to study the effects of RNAi suppression in mosquitoes [13-16].

This report describes the production of a recombinant SINV that expresses a heterologous VSR protein and use of the virus to directly study the effects of RNAi on mosquito infection. A TE/3'2J virus was engineered to express the B2 protein, with the hypothesis that expression of B2 during SINV infection would inhibit the RNAi response in infected mosquito cells and that this inhibition will lead to increased virus replication within the mosquito. The VSR was functional in mosquito cells and affected the replication of TE/3'2J virus in *Ae. aegypti *cell culture. Mosquito infection experiments show that not only are rates of infection and dissemination of SINV in *Ae. aegypti *increased if RNAi is inhibited, but that the B2-expressing virus became highly pathogenic in the mosquito, significantly shortening the mosquitoes' lifespan. These studies highlight the necessity for RNAi from both the standpoint of mosquito survival and arbovirus persistence.

## Results

### Inhibition of RNAi by a SINV-expressed VSR

**Figure 1:**
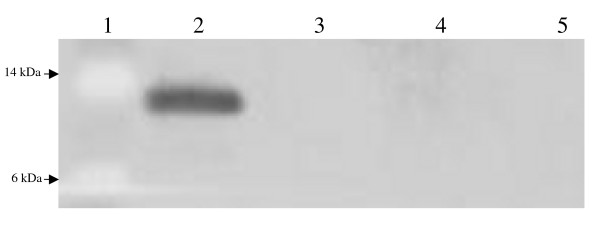
**Detection of V5-B2 protein in mosquito cell culture**. V5-B2 protein was detected by immunoblot using anti-V5 antibody in total protein from Aag2 cell culture. Molecular weights are indicated on the left side of each panel. Lane 1, protein molecular weight marker. Lane 2, TE/3'2J/B2-infected cells. Lane 3, TE/3'2J/GFP-infected cells. Lane 4, TE/3'2J-infected cells. Lane 5, Mock-infected cells.

After rescue of infectious virus from cDNA-derived RNA, expression of V5 epitope-tagged B2 protein from the second subgenomic promoter was verified by immunoblot analysis of total protein from infected Aag2 cells. Using a commercial antibody against the V5 epitope, we observed a single band of approximately 12 kilodaltons (kDa) in B2-infected cells (Figure 1), in agreement with the predicted size of V5-tagged B2 protein (12.4 kDa). No bands were detected in cells infected with TE/3'2J, TE/3'2J/GFP, or mock-infected cells.

**Figure 2:**
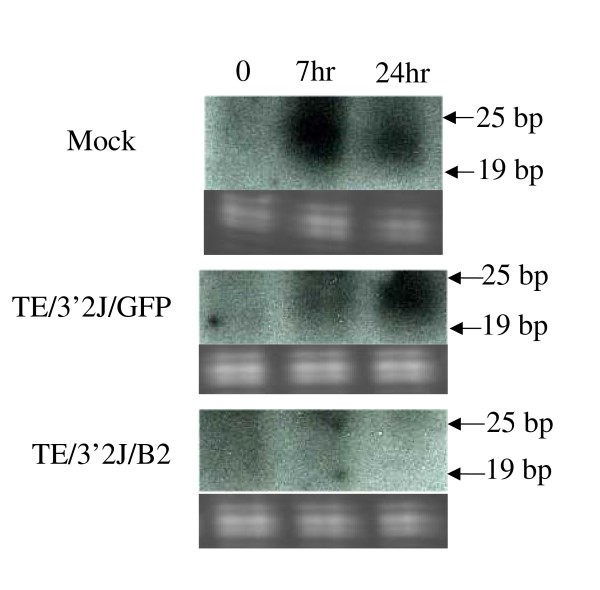
**Accumulation of Dicer cleavage products in cells infected with TE/3'2J/GFP or TE/3'2J/B2 virus**. Cell lysates were generated from Aag2 cells 36 hours post mock-, TE/3'2J/GFP, or TE/3'2J/B2 virus-infection (MOI = 0.01) (indicated to left of each panel). A synthetic 500 bp biotinylated dsRNA product was introduced into the lysates and, at indicated time points, samples were taken and the presence of small RNAs was determined by Northern blot analysis. Ethidium bromide-stained ribosomal RNAs located below each blot serve as loading controls. Arrows indicate position of 25 and 19 nucleotide markers.

To determine the ability of SINV-expressed B2 protein to inhibit the mosquito RNAi response, an *in vitro *dicing assay was performed. A synthetic 500 bp biotinylated dsRNA derived from the bacterial β-galactosidase gene was introduced into Aag2 cell lysates produced from cells mock-infected or infected with GFP- or B2-expressing virus. The presence of siRNA product suggested that dsRNA processing to siRNAs occurs in Aag2 cells. Biotinylated RNA approximately 21–23 nucleotides in length accumulated in mock- and TE/3'2J/GFP virus-infected cell lysates, whereas little biotinylated RNA was detected in the expected size range at any time points tested in TE/3'2J/B2 virus-infected cell lysates (Figure 2).

**Figure 3:**
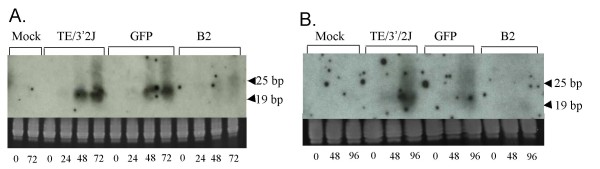
**Detection of virus-specific siRNAs in Aag2 cells (A) and *Ae. aegypti *(Higgs White Eyes) mosquitoes (B)**. Monolayers of Aag2 cells were mock infected or infected with TE/3'2J, TE/3'2J/GFP, or TE/3'2J/B2 virus at MOI = 0.01. Mosquitoes were intrathoracically inoculated with cell culture medium from TE/3'2J, TE/3'2J/GFP, or TE/3'2J/B2 virus. At indicated times post infection, total RNA was isolated and probed using an E1-specific riboprobe for virus-derived siRNA. Ethidium bromide-stained ribosomal RNA below each blot serves as a loading control. Time in hours post infection is noted below ribosomal RNA controls. Arrows indicate position of 25 and 19 nucleotide markers.

After determining that B2 protein could inhibit the accumulation of siRNAs derived from a synthetic dsRNA in cell culture-derived lysates, we investigated the ability of the protein to inhibit virus-specific siRNA accumulation during virus replication in mosquito cells. The accumulation of SINV E1 gene-derived antisense small RNAs was examined in infected Aag2 cells over a 72-hour time course. Beginning at 24 hours and continuing to 72 hours post-infection, SINV-specific RNAs 21–23 nucleotides in size were detected in Aag2 cells infected with TE/3'2J and TE/3'2J/GFP viruses. The size of the small RNAs is consistent with previous reports of virus-derived siRNAs detected in mosquito cells [6,17-21]. Few RNAs of this size were detected at any time in mock-infected cells or cells infected with TE/3'2J/B2, suggesting that B2 protein can function to inhibit virus-specific RNAi in mosquito cell culture (Figure 3A).

The same methodologies were used to detect virus-derived siRNAs in intrathoracically-injected *Ae. aegypti *mosquitoes. Similar to cell culture, small RNAs 21–23 nucleotides in size were detected in TE/3'2J- and TE/3'2J/GFP-infected mosquitoes at 48 hours post-infection (Figure 3B). siRNAs accumulated to large amounts by 96 hours in mosquitoes infected with TE/3'2J, and to a lesser extent in TE/3'2J/GFP-injected mosquitoes. No virus-specific siRNAs could be detected in mosquitoes mock-injected with cell culture medium or injected with TE/3'2J/B2, indicating that B2 protein could inhibit targeted degradation of the SINV genome in the context of infected mosquitoes (Figure 3B).

### Effects of B2 protein expression on SINV replication

**Figure 4:**
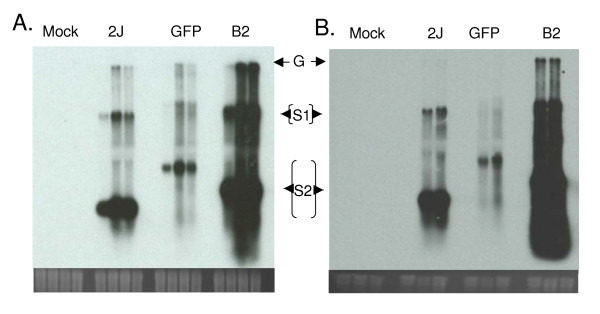
**Detection of viral RNAs in Aag2 cells (A) and *Ae. aegypti *mosquitoes (B)**. Monolayers of Aag2 cells were mock-infected or infected with TE/3'2J, TE/3'2J/GFP, or TE/3'2J/B2 virus at MOI = 0.01. Mosquitoes were intrathoracically-inoculated with cell culture medium, TE/3'2J, TE/3'2J/GFP, or TE/3'2J/B2 virus. At indicated times post infection, total RNA was isolated and an E1-specific riboprobe was used to detect  virus genomic and subgenomic RNA. Ethidium bromide-stained ribosomal RNA below each blot serves as a loading control. Time post infection for each virus in (A) is 0, 24, 48, and 72 hrs, and in (B) 0, 48, and 96 hrs. G = genomic; S1 = first subgenomic; S2 = second subgenomic.

The inhibition of siRNA accumulation showed that B2 protein could inhibit RNAi in mosquito cells. To determine the effects that RNAi inhibition may have on SINV replication, we first examined the ability of SINV RNA to accumulate in infected cells. Using the same total RNA samples used for siRNA detection, we examined the accumulation of viral genomic and subgenomic RNA species in Aag2 cells and mosquitoes by Northern blot analysis (Figure 4A and 4B). Starting at 24 hours post-infection, three viral RNA species were detected in cells infected with TE/3'2J, TE/3'2J/GFP, and TE/3'2J/B2 viruses. These bands represent the genomic, first subgenomic, and second subgenomic RNAs produced during virus infection. The second subgenomic RNA, expressed from the most 3' virus promoter, is the most highly transcribed RNA species for all three viruses, consistent with previous reports [22]. The observed inhibition of siRNA accumulation in TE/3'2J/B2-infected cells corresponded with a distinct increase in viral RNA accumulation. Considerably more viral RNA was detected in cells and mosquitoes infected with TE/3'2J/B2 virus beginning at 24 hours post-infection and continuing throughout all time points tested. Much less viral RNA accumulated in TE/3'2J/GFP-infected cells and mosquitoes, an expected outcome considering the increase in genome size and accompanying decrease in replication efficiency [23]. No bands were observed in RNA from mock-infected cells.

**Figure 5:**
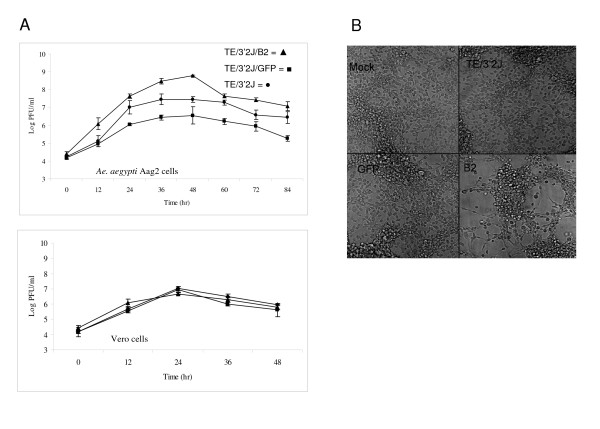
**Growth of TE/3'2J, TE/3'2J/GFP, and TE/3'2J/B2 viruses in invertebrate and vertebrate cells**. **A) **Triplicate flasks containing cell monolayers of Aag2 cells (A, top panel) and Vero cells (A, bottom panel) were infected at MOI = 0.01. Titers were determined by plaque formation on Vero cells. Black circles = TE/3'2J, Black squares = TE/3'2J/GFP, Black triangles = TE/3'2J/B2. **B) **Cytopathic effect of TE/3'2J, TE/3'2J/GFP, and TE/3'2J/B2 on Aag2 cells at 72 hrs post infection (MOI = 0.01).

Because siRNA accumulation was inhibited and viral RNA amounts increased in TE/3'2J/B2 virus-infected cells, we tested if suppression of RNAi by B2 would cause more infectious virus to be produced during infection. We performed two-step growth curve analysis in Aag2 and Vero cells to determine the effects of B2 protein expression on infectious virus production (Figure 5A). Virus titers were determined from supernatant of cells infected with TE/3'2J, TE/3'2J/GFP, and TE/3'2J/B2 viruses at a multiplicity of infection (MOI) = 0.01. TE/3'2J/B2 replicated to a maximum titer of 8.8 log_10 _PFU/ml at 48 hours post-infection in Aag2 (Figure 5, top panel). This was more than 10-fold higher than TE/3'2J (7.4 log_10 _PFU/ml) and 100-fold higher than TE/3'2J/GFP (6.6 log_10 _PFU/ml). TE/3'2J/GFP replicated less efficiently than TE/3'2J, suggesting that virus encoding an insert may be less able to replicate in Aag2 cells. A marked decrease in titer was observed at later time points during TE/3'2J/B2 virus infection of Aag2, coinciding with the presence of cytopathic effects not observed in TE/3'2J- or TE/3'2J/GFP-infected cells (Figure 5B). Notwithstanding, the titer of TE/3'2J/B2 virus was greater than the titers of TE/3'2J and TE/3'2J/GFP at all time points tested in this cell line.

Growth curve analysis was also performed in Vero cells to determine the effects of B2 protein expression on SINV replication in vertebrate cells (Figure 5A, bottom panel). Surprisingly, replication of all three viruses was similar in this cell line. Peak titers of 7.1, 7.0, and 6.7 log_10 _PFU/ml were reached at 48 hours post-infection for TE/3'2J, TE/3'2J/GFP, and TE/3'2J/B2 viruses, respectively. The similar replication kinetics observed for all three viruses suggests that RNAi may not be as important for antiviral immunity in vertebrate cells compared to mosquito cells.

**Figure 6:**
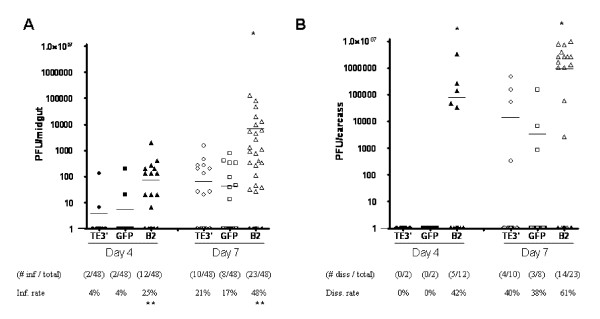
**Infection and dissemination of TE/3'2J, TE/3'2J/GFP, and TE/3'2J/B2 viruses in *Ae. aegypti *mosquitoes following oral bloodmeal**. At the indicated day post-bloodmeal, viral titers were determined for **A) **midguts and remaining **B) **mosquito carcass. n = 48 per group. "TE/3"' = TE/3'2J, "GFP" = TE/3'2J-GFP, "B2" = TE/3'2J/B2. Horizontal line represents the mean for each data set. (*) above data set indicates that the mean TE/3'2J/B2 titer is significantly higher than TE/3'2J and TE/3'2J/GFP infections. (**) below the infection and dissemination rates indicates significantly higher infection and dissemination rates as compared to TE/3'2J virus infection. Due to the lack of dissemination positive mosquitoes in the Day 4 TE/3 and GFP samples (Figure B), statistical significance of the Day 4 B2 group as compared to the TE/3 and GFP groups could not be determined.

Based on our data showing increased replication of TE/3'2J/B2 in Aag2 cells, we tested whether TE/3'2J/B2 would increase virus replication in mosquitoes following an infectious oral bloodmeal. At four and seven days post infection (dpi), midguts were dissected from 48 mosquitoes per group and, along with remaining mosquito carcasses, were titrated on Vero cells. Titers of infectious virus represent the extent to which virus replicated in individual mosquitoes while the total number of infected midguts and carcasses represent the infection and dissemination rates, respectively (Figure 6). Because electroporation-derived recombinant SINVs and invertebrate cell-derived viruses produced from TE/3'2J inefficiently infect mosquito midguts following oral infection, virus was passed once in Vero cells prior to use in blood feeds [24,25]. TE/3'2J/B2 virus exhibited the highest rates of infection and dissemination and the highest average titers at both time points. Of 48 mosquitoes tested, 12 (25%) had detectable TE/3'2J/B2 virus in the midgut at four dpi, significantly more compared to TE/3'2J and TE/3'2J/GFP (P = 0.0074 for both comparisons, Fisher's exact test). Infection rates increased in all three groups at seven dpi, but the number of TE/3'2J/B2 virus-infected mosquitoes remained significantly higher than TE/3'2J (P = 0.0094) and TE/3'2J/GFP (P = 0.0020). TE/3'2J and TE/3'2J/GFP virus infection rates did not differ significantly at four or seven dpi. All mosquitoes exhibiting a disseminated infection had detectable virus in the midgut. Five of 12 mosquitoes (42%) with detectable TE/3'2J/B2 virus in the midgut exhibited disseminated infection at day four while no virus was detected in carcasses of mosquitoes infected with TE/3'2J or TE/3'2J/GFP virus. At seven dpi, 61% (14 of 23) of TE/3'2J/B2 virus-infected mosquitoes had disseminated infections, as compared to 40% (4 of 10) for TE/3'2J- and 38% (3 of 8) for TE/3'2J/GFP-infected mosquitoes. Significantly higher average TE/3'2J/B2 virus titers were found in the midgut at seven dpi (P = 0.0446 TE/3'2J:TE/3'2J/B2; P = 0.0439 TE/3'2J/GFP:TE/3'2J/B2; unpaired Student's t test) and in mosquito carcasses at seven dpi (P = 0.0043 TE/3'2J:TE/3'2J/B2; P = 0.0038 TE/3'2J/GFP:TE/3'2J/B2). Average TE/3'2J/B2 titers in the midgut at four dpi were not statistically higher (P = 0.1023 TE/3'2J:TE/3'2J/B2, P = 0.1115 TE/3'2J/GFP:TE/3'2J/B2). At four and seven dpi, infection and dissemination titers were not statistically different between TE/3'2J and TE/3'2J/GFP viruses.

### Ae. aegypti mortality associated with TE/3'2J/B2 virus infection

**Figure 7:**
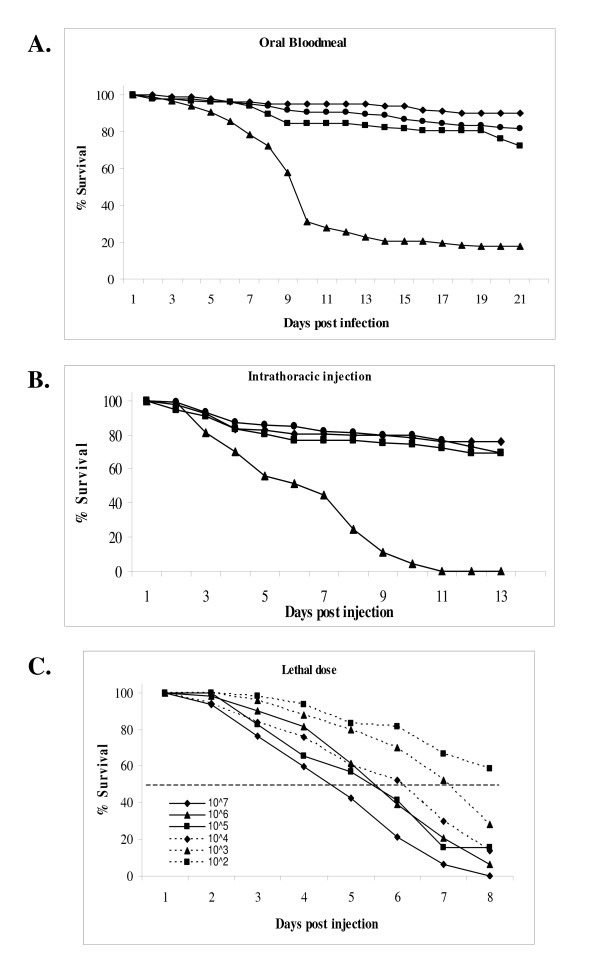
**Virus associated mortality of *Ae. aegypti *HWE mosquitoes following infection by TE/3'J/B2 virus**. **A) Oral bloodmeal infection **Mosquitoes were given an infectious oral bloodmeal containing 1 × 10^7 ^PFU of virus and kept at optimal rearing conditions. Mortality was monitored daily for a total of 21 days. n = 200 mosquitoes per group. **B) Infection via intrathoracic injection **Mosquitoes were injected with virus stock diluted to 1 × 10^7 ^PFU/ml and mortality was monitored daily. Day one mortality was not included. Black diamonds = Mock; Black circles = TE/3'2J; Black squares = TE/3'2J/GFP; Black triangles = TE/3'2J/B2. **C) Determination of a mosquito 50 percent lethal dose for TE/3'2J-B2 infection**. Groups of mosquitoes were intrathoracically injected with TE/3'2J/B2 virus diluted ten-fold and mortality was monitored daily. n = 50 mosquitoes/group. White bar indicates 50% mortality.

Mosquito mortality assays were performed to determine the effects of virus infection on mosquito survival. From observations made during determination of infectious virus titers in orally infected mosquitoes, we predicted that TE/3'2J/B2 virus was able to kill mosquitoes more effectively than TE/3'2J or TE/3'2J/GFP. Female mosquitoes were given a bloodmeal containing 1 × 10^7 ^PFU/ml of TE/3'2J, TE/3'2J/GFP, TE/3'2J/B2, or cell culture medium only. Engorged females were separated and kept at optimal rearing conditions, including fresh sugar and water daily for 21 days, and individual mortality was monitored daily. Beginning at four dpi, more mosquitoes infected with TE/3'2J/B2 virus died than mock-infected mosquitoes or those infected with TE/3'2J and TE/3'2J/GFP viruses. Eighty-three percent of the mosquitoes ingesting a bloodmeal containing TE/3'2J/B2 were dead by day 21 versus 21% for mock, 11% for TE/3'2J, and 30% for TE/3'2J/GFP exposed mosquitoes (Figure 7A). Daily survival for mosquitoes that ingested TE/3'2J/B2 virus was significantly lower than mock, TE3'2J, or TE/3'2J/GFP-infected mosquitoes (P < 0.0001 for each comparison, Logrank test). Survival of TE/3'2J-infected mosquitoes was significantly different from TE/3'2J/GFP-infected mosquitoes (P = 0.0030). Survival of mosquitoes infected with TE/3'2J and TE/3'2J/GFP was not significantly different from mock-infected mosquitoes (P = 0.0623 and 0.2496, respectively).

Because some mosquitoes that ingested a bloodmeal may not have become infected, individual mosquitoes were intrathoracically injected with virus to more accurately correlate infection with mortality. Female mosquitoes were injected with approximately 700 PFU of virus or cell culture medium and were monitored daily for mortality. At ten days post-infection, all mosquitoes injected with TE/3'2J/B2 virus were dead, whereas by day 13, at least 70% of mock-, TE/3'2J-, and TE/3'2J/GFP-injected mosquitoes survived (Figure 7B), suggesting that TE/3'2J/B2 virus infection caused the observed mortality in *Ae. aegypti *mosquitoes.

To determine if TE/3'2J/B2-associated mortality was dose-dependent, a 50% lethal dose at seven days post-injection was determined by mosquito intrathoracic injection (Figure 7C). Groups of 50 mosquitoes were injected with TE/3'2J/B2 virus diluted 10-fold in cell culture medium and monitored for mortality. TE/3'2J/B2 infection was extremely lethal, needing less than one PFU per mosquito to cause more than 50% mortality, and was dose-dependent. The median survival time for mosquitoes was five days at the highest dose (10^7 ^PFU/ml) and seven days at the lowest dose that caused more than 50% mortality (10^3 ^PFU/ml).

**Figure 8:**
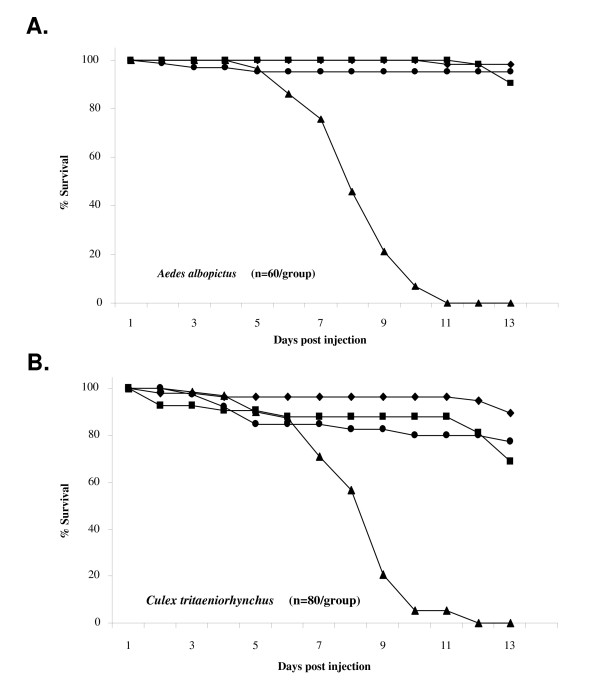
**Virus-associated mortality in different mosquito species**. Female *Ae. albopictus *(A) or *Cx. tritaeniorhynchus *(B) mosquitoes were injected with virus stock diluted to 1 × 10^7 ^PFU/ml and mortality was monitored daily. Day one mortality was not included. Black diamonds = Mock; Black circles = TE/3'2J; Black squares = TE/3'2J/GFP; Black triangles = TE/3'2J/B2.

SINV is capable of infecting many different species of mosquitoes, so TE/3'2J/B2 virus-associated mortality following intrathoracic injection was also tested in *Aedes albopictus *and *Culex tritaeniorhynchus *mosquitoes (Figure 8). Female mosquitoes were injected with approximately 700 PFU of virus or cell culture medium as a mock-infected control and mortality was monitored daily. In both mosquito species, TE/3'2J/B2 virus killed 100% of injected mosquitoes by 11–12 days post-injection. Little mortality was observed in mock-, TE/3'2J-, or TE/3'2J/GFP- injected *Ae. albopictus *mosquitoes (Figure 8A). Interestingly, *Cx. tritaeniorhynchus *mosquitoes injected with TE/3'2J or TE/3'2J/GFP survived less well than mock infected mosquitoes (Figure 8B).

## Discussion

RNAi is a major antiviral response in mosquitoes. The only other described mosquito immune response to arbovirus infection is mediated by the Toll antimicrobial pathway [26]. RNAi is a highly conserved mechanism that is stimulated by the presence of an invading virus and controls viral replication through the sequence-specific degradation of the virus RNA. To study RNAi during SINV infection of *Ae. aegypti*, we have engineered a double subgenomic SINV to express B2 protein, a potent VSR [13]. In a recently published study, SINV-B2 and ONNV-B2 were shown to cause mortality in injected *Ae. aegypti *and *An. gambiae *mosquitoes, respectively [10]. We show that mosquitoes infected in a more natural manner (*per os*) with a B2 expressing SINV demonstrate increased viral titers, higher levels of viral dissemination from the midgut, and greatly enhanced virus-induced mortality in *Ae. aegypti*, *Ae. albopictus*, and *Cx. tritaeniorhynchus *mosquitoes. In our system, the B2 protein is translated only in infected cells, avoiding potential off-target effects associated with transient dsRNA-mediated silencing of the RNAi pathway. Tschuch et al found that introduction of siRNA specific for green fluorescent protein (GFP) into human cells that did not express GFP non-specifically perturbed expression of more than 200 genes [27]. A similar non-specific dsRNA-mediated regulation of gene expression has been described in sandfly (*Lutzomyia longipalpis*) cell culture and the marine shrimp, *Litopenaeus vannamei *[28,29]. Although similar experiments have not been performed in mosquito cells, introduction of dsRNA could have a similar effect. Detectable, yet not statistically-significant increases in viral titer have been observed when control experiments injecting β-gal dsRNA and virus into mosquitoes have been performed [7,30]. While the potential for non-specific response of the mosquito immune system to dsRNA is intriguing and should be studied further, examining the RNAi response using a SINV expressing a viral RNAi suppressor is superior to dsRNA-mediated interference of the RNAi pathway.

Three lines of experimental evidence suggest that the B2 protein was functional in RNAi suppression when expressed during TE/3'2J/B2 virus infection. First, *in vitro *dicing experiments show inhibition siRNA accumulation in cell lysates derived from TE/3'2J/B2 virus-infected Aag2 cells. The presence of B2 protein inhibits the accumulation of biotinylated siRNAs, presumably by binding to the synthetic dsRNA and sequestering from Dicer-2. The presence of siRNAs in mock- and TE/3'2J/GFP-infected lysates provides evidence that Aag2 cells have a functional RNAi mechanism. Also, this shows that inhibition of siRNA accumulation is specific to TE/3'2J/B2 virus infection. The second line of evidence comes from Northern blot analysis of small RNAs in mosquito cells. Considerably less SINV-specific siRNAs accumulated in cell culture and mosquitoes infected with TE/3'2J/B2 virus compared to TE/3'2J and TE/3'2J/GFP virus infection. The dsRNA formed by viral replicative intermediates may be bound by B2 protein, protecting the dsRNA from detection by the RNAi machinery.

Finally, virus titers observed in Aag2 cells and adult *Ae. aegypti *mosquitoes were much higher when B2 protein was expressed during infection. This agrees with previous data showing that inhibition of the RNAi pathway allows for arboviruses to replicate more efficiently in mosquitoes [6,7]. By injecting mosquitoes with dsRNA targeting Dicer-2 or Argonaute-2 after an infectious bloodmeal, Campbell et al [6] were able to show that SINV titers in individual mosquitoes increased significantly by day four as compared to β-gal dsRNA injected controls. The same effect was not seen at day seven and the authors suggest this may be due to a stimulation of the antiviral response by this time point or degradation of the dsRNA triggers via decay [6]. A similar general phenomenon was seen with ONNV infection of *An. gambiae *mosquitoes, with a detectable increase in virus titer up to six days post infection [7]. This difference may be explained by the inoculation route as both dsRNA and ONNV were administered intrathoracically, bypassing any infection barriers associated with the midgut and ensuring introduction of virus and dsRNA into the hemocoel [7]. A significant increase in SINV titers was observed at both four and seven days post infectious bloodmeal in mosquitoes ingesting TE/3'2J/B2 virus. The RNAi response is continuously inhibited by B2 protein as it is produced in infected mosquito cells. dsRNA intermediates or secondary structure of the virus genome will not be recognized by the RNAi machinery, allowing virus replication to continue unabated.

Our data indicate that SINV becomes pathogenic to mosquitoes when RNAi is suppressed during virus infection. Pathology and mortality have been associated with alphavirus infection of mosquitoes, but the pathology is specific to the midgut or salivary glands and the mortality can, in some cases, be attributed to intrathoracic inoculation of large amounts of virus [1-4]. TE/3'2J/B2 virus-associated mortality was infection route- and mosquito species independent: significantly more *Ae. aegypti *died when exposed to TE/3'2J/B2 virus either orally or via injection and *Ae. albopictus *and *Cx. tritaeniorhynchus *were susceptible to TE/3'2J/B2 virus following intrathoracic injection.

We originally hypothesized that the observed mortality was caused by apoptotic death of a majority of infected cells in the mosquito. FHV has been shown to induce apoptosis in *Drosophila *cell culture through the depletion of an intracellular inhibitor of apoptosis [31]. Apoptosis in alphavirus-infected mosquito cell lines is dependent on the amount of viral RNA and infectious virus produced during infection [32-35]. We show that considerably more SINV subgenomic RNA and 100-fold more infectious virus are produced in mosquitoes when B2 protein is expressed during infection. However, apoptosis could not be detected within infected cells in sections of virus-infected mosquitoes (data not shown). It is possible that cell death caused by TE/3'2J/B2 virus is via a non-apoptotic mechanism. Necrosis has been observed in midgut epithelial cells of *Culiseta melanura *mosquitoes orally-infected with eastern equine encephalitis virus at times corresponding to peak midgut virus titers [1]. Electron microscopy of infected cell morphology and detailed analysis of infected mosquito gene expression using microarray analysis may help to more clearly define the mechanism of TE/3'2J/B2 virus-associated mortality.

Behavioral changes have been suggested as a direct result of arbovirus infection [1]. TE/3'2J/B2 virus infection of the brain and sensory organs may lead to changes in mosquito behavior that could eventually lead to death such as decreased nutrient and water uptake or inability to oviposit. Although not examined here, quantitative observation of behaviors such as blood feeding and oviposition may provide evidence for neurological effects associated with virus infection [36].

The salivary glands are an important organ for successful transmission of arboviruses. If TE/3'2J/B2 virus infection leads to cytopathology in the salivary glands, transmission of the virus may be more efficient or could be hindered. It was suggested that SINV-associated pathology in *Ae. albopictus *midgut-associated musculature and salivary glands could lead to a decrease in feeding success [4]. If this is true, then transmission of TE/3'2J/B2 virus could be more efficient as mosquitoes take a longer time to probe the skin prior to imbibing blood. However, if salivation were compromised by virus-induced cytopathology, transmission of virus from the salivary glands would be less efficient due to decreased saliva inoculation volumes.

The B2 protein alone is likely not the mosquito mortality-associated factor. FHV was capable of replication to high titers when injected into *Ae. aegypti *mosquitoes, but no mortality was associated with the infection [37]. Also, transgenic *Drosophila *flies that express B2 protein have been shown to be deficient in siRNA-mediated but not microRNA-mediated RNA silencing and are more susceptible to RNA virus infection and virus-associated mortality [16,38]. This suggests that B2 protein by itself is not capable of causing mortality in dipterans, but that B2 protein in combination with an infecting RNA virus is capable of protecting virus replication from the influence of RNAi. Additionally, recent experiments show that a SINV expressing a B2 mutant incapable of binding siRNAs does not suppress RNAi in mosquitoes [10], indicating that the siRNA binding activity of B2 is responsible for the effect observed in our experiments.

The implications of TE/3'2J/B2 virus-associated mortality are two-fold. First, unlike pathogenic viruses that do not require persistent infection of the host, arboviruses may not encode true suppressors of RNAi. B2 protein and many proteins produced by pathogenic plant viruses are dsRNA binding proteins and potent suppressors of the RNAi response. The dsRNA-binding protein NSs of La Crosse virus, an arbovirus transmitted by *Ochlerotatus triseriatus *mosquitoes, was initially suggested to be a VSR in mammalian cells, but was later shown to be an interferon antagonist that did not interfere with RNAi in mosquito cells [39,40]. Similar conclusions were made with the NS1 protein of influenza A virus, a non-vectored virus [41,42]. To our knowledge, there has been no description of an arbovirus-produced protein that is a VSR in mosquito cells, and our data suggest that encoding a VSR may be detrimental to arbovirus transmission.

Second, mortality of TE/3'2J/B2 virus-infected mosquitoes suggests there may be a delicate balance between mosquito immune response and virus replication that allows for the persistent nature of arbovirus infection in the vector. In the model of Semliki Forest virus (genus *Alphavirus*) regulation of RNA replication, production of negative-strand RNA, that serves as a template for full-length virus genome and subgenomic RNA, is restricted to the early phase of replication [43]. Limiting the production of negative-strand RNA may allow for more efficient allocation of cellular resources to progeny virus production and may have evolved to exclude subsequent viruses from establishing infection. It was proposed that regulation of negative-strand RNA synthesis, in turn regulating full length and subgenomic positive-strand RNA, evolved to moderate virus-associated virulence in the mosquito vector [43]. Our experiments with TE/3'2J/B2 virus suggest that the replicase proteins of SINV, which control the amounts of viral RNA through sequential cleavage of polyprotein complexes, may not be the sole regulators of virus RNA quantities. Rather, there appears to be a balanced effort from both the virus and the mosquito immune system to regulate replication so that the virus can persist in mosquitoes without causing significant adverse effects, allowing the virus to increase its transmission efficiency to a new host. Further studies could compare inhibition of siRNA accumulation at early times during TE/3'2J and TE/3'2J/B2 virus infection and may shed light on the potential cooperation of viral replicase complexes and RNAi response in regulation of virus RNA production in mosquito cells. Identifying key mosquito factors necessary for viral RNA regulation may lead to novel transgenic mosquitoes that over-express these factors and are, therefore, refractory to arbovirus infection.

## Conclusion

Alphaviruses must be transmitted between insect and vertebrate hosts to be maintained in nature, and thus must optimize their transmission potential in each host to ensure continuity. Disruption of this optimization in mosquitoes adversely affects the ability of the mosquito to control infection and results in death of the mosquito, which will reduce the fitness of the virus over time. Thus it appears that alphaviruses have developed a delicate balance between robust replication and limited pathology in their mosquito hosts that allows for persistent infection and efficient vectoring.

## Methods

### Cells and mosquitoes

African green monkey kidney (Vero) and baby hamster kidney (BHK-21) cells were maintained in minimal essential medium (MEM) supplemented with 7% fetal bovine serum (FBS), 1× nonessential amino acids for MEM (NEAA), 2 mM L-glutamine, 100 units/ml penicillin and 100 μg/ml streptomycin and were grown at 37°C with 5% CO_2_. *Ae. aegypti *Aag2 cells were maintained in modified Schneider's *Drosophila *medium supplemented with 10% FBS, L-glutamine, and antibiotics and were grown at 28°C with ambient CO_2_.

*Ae. aegypti *Higgs white eye (HWE) mosquitoes, a variant of the Rexville D strain originating from Puerto Rico (Division of Vector-borne Infectious Diseases, Centers for Disease Control and Prevention (CDC), Fort Collins, CO) [20,44,45] were reared at 28°C, 80% relative humidity, with a 16:8 light:dark photoperiod. Sugar and water sources were provided *ad libitum*. *Ae. albopictus *mosquitoes originating from Lake Charles, Louisiana (CDC) and *Cx. tritaeniorhynchus *mosquitoes originating from Thailand (provided by Dr. Barry Miller, CDC) were reared at 27°C, 80% relative humidity, with a 14:10 light:dark photoperiod.

### Viruses

Construction of the plasmid infectious cDNA clones pTE/3'2J and pTE/3'2J encoding green fluorescent protein (GFP) have been previously described [46,47]. To construct pTE/3'2J/B2, the 321 base pair B2 gene was amplified by polymerase chain reaction (PCR) from an expression plasmid containing the entire B2 gene. The forward primer contained sequence of V5 epitope, encoding the C-terminal 14 amino acids (GKPIPNPLLGLDST) of V protein from simian virus 5 (family *Paramyxoviridae*) [48]. The PCR product was digested with Xba I restriction endonuclease and ligated into the Xba I site of pTE/3'2J. Proper insertion of V5-B2 was verified through orientation PCR and sequencing.

Infectious virus was produced by electroporation of linearized plasmid as described previously [46,47]. Electroporations were performed in BHK-21 cells and each virus was passaged once in Vero cells. All viruses were aliquoted, titrated using standard assays, and maintained at -80°C until use.

### Immunoblot analysis

For immunoblot analysis, cell monolayers were infected with TE/3'2J, TE/3'2J/GFP, and TE/3'2J/B2 virus at a MOI~0.01, or mock-infected with medium. Forty-eight hours post-infection, medium was removed and cells were scraped into PBS containing protease inhibitors (Roche Applied Science, Indianapolis, IN). Cell suspensions were sonicated and stored at -20°C. Ten micrograms of total protein were separated by SDS-polyacrylamide gel electrophoresis in a 10% gel and transferred to a nitrocellulose membrane at 30 volts. Membranes were blocked for 1 hour at room temperature in PBS plus 0.05% Tween-20 (PBS-T) and 5% lowfat dry milk (blocking buffer). V5-B2 protein was detected by incubating membranes at 4°C overnight with a mouse anti-V5 IgG antibody (Invitrogen Corporation, Carlsbad, CA) diluted 1:5,000 in blocking buffer followed by a room temperature incubation with a horseradish peroxidase-conjugated goat anti-mouse IgG secondary antibody (KPL, Inc., Gaithersburg, MD) diluted 1:1,000 in blocking buffer for 30 minutes. The Pierce ECL western detection kit (Thermo Fisher Scientific, Inc., Rockford, IL) was used to develop the membranes according to manufacturer's protocols. Chemiluminescence was detected using the Storm 860 phosphoimager (Molecular Dynamics, Inc., Sunnyvale, CA).

### In vitro dicing assay

Cell-free lysates were generated from Aag2 cells that were mock-infected or infected with TE/3'2J, TE/3'2J/GFP, or TE/3'2J/B2 virus (MOI: 0.01). Lysates were formed 36 hours post-infection using a protocol modified from Haley et al [49]. Briefly, cells were washed three times in PBS and resuspended in 1× lysis buffer (100 mM potassium acetate; 30 mM Hepes-KOH, pH 7.4; 2 mM magnesium acetate) with protease inhibitors and 5 mM DTT. The cells were disrupted in a Dounce homogenizer and centrifuged at 14,000 × g for 25 minutes at 4°C. The supernatant was flash frozen in a dry ice/ethanol bath and stored at -80°C. Dicing activity reactions were constituted as described previously [49] and incubated at 28°C. Each reaction contained 1/2 volume of cell lysate (normalized for protein concentration), 1/3 volume of 40× reaction mix (50 μl water; 20 μl 500 mM creatine monophosphate; 20 μl amino acid stock at 1 mM each, 2 μl 1 M DTT, 2 μl 20 U/μl RNasin, 4 μl 100 mM ATP, 1 μl 100 mM GTP, 6 μl 2 U/μl creatine phosphokinase, 16 μl 1 M potassium acetate) and 450 ng of 500 bp biotinylated β-gal dsRNA [49]. Biotinylated dsRNA was produced by *in vitro *transcription of a 500 base pair region of the β-galactosidase gene from *Escherichia coli *using forward and reverse primers containing a bacteriophage T7 bacteriophage polymerase promoter. Transcribed RNA products were treated with DNase, extracted once with phenol/chloroform, once with chloroform, and precipitated in ethanol. At each timepoint, 10 μl of the dicing reaction were removed, added to 2× proteinase K buffer (200 mM Tris-Cl, pH 7.5; 25 mM EDTA, pH 8.0; 300 mM NaCl; 2% weight/volume sodium dodecyl sulfate) and flash frozen. RNA was extracted using phenol/chloroform followed by a chloroform isoamyl alcohol extraction and precipitated in ethanol. RNA was electrophoresed on a 20% non-denaturing polyacrylamide gel, stained with ethidium bromide, electrophoretically transferred to a BrightStar membrane (Ambion, Inc., Austin, TX.) and UV-crosslinked. Biotinylated RNA was detected with the BrightStar BioDetect Kit (Ambion, Inc.) and exposed to autoradiography film for approximately 1.5 hours.

### Growth curve analysis

For growth curve analysis, triplicate monolayers of Aag2 and Vero cells in 25 cm^2 ^flasks were infected with virus at an MOI ~0.01. Immediately following infection, a 500 μl sample was taken to determine input virus titer and an additional 500 μl of fresh growth medium was reintroduced. Removal and addition of medium procedures were conducted every 12 hours post-infection for a total of 48 hours for Vero cells or 84 hours for Aag2 cells. Samples were immediately stored at -80°C until determination of titers by plaque titration.

### Detection of virus-specific RNA

Virus-specific RNA species (genomic, subgenomic, and siRNAs) in cell culture and whole mosquitoes were detected by Northern blot analysis. For the detection of viral RNA, Aag2 cells were infected as described for virus growth curves. At 0, 24, 48, and 72 hours post-infection, total RNA was extracted from cells using Trizol reagent (Invitrogen Corp.) following the manufacturer's recommended protocols. For viral RNA detection from infected mosquitoes, 3 to 5 day old female mosquitoes were injected with 69 nl of 1 × 10^7 ^PFU/ml of virus, or mock-injected with medium using a Nanoject II auto-nanoliter injector (Drummond Scientific Company, Broomall, PA). Immediately following injection and at day two and day four post-infection, ten individual mosquitoes from each experimental group were triturated in 500 μl of Trizol reagent and total RNA was extracted according to manufacturer's protocols.

Twenty micrograms (for Aag2 cells siRNA detection) or 40 μg (for mosquito siRNA detection) of RNA per sample were used for SINV-specific siRNA detection. Low molecular weight RNAs were separated by electrophoresis in a 15% denaturing polyacrylamide gel stained with ethidium bromide to visualize concentrations of RNA as a loading control. RNA was transferred to a neutral-charged nylon membrane and chemically cross-linked using 1-ethyl-3-(3-dimethylaminopropyl) carbodiimide (EDC) [50]. Membranes were pre-hybridized in Ultrahyb buffer (Ambion, Inc.) at 42°C for 30 minutes. A sense-specific biotinylated single-stranded RNA probe corresponding to antisense nucleotides 10,765 to 11,264 (E1 gene) of TE/3'2J was generated from a PCR template containing a T7 promoter. The MEGAscript *in vitro *transcription kit (Ambion, Inc.) was used according to manufacturer's recommended protocols with 9% of the total UTP conjugated to biotin. Five micrograms of riboprobe were reduced to 50–100 nt fragments by hydrolysis in 200 mM carbonate buffer at 60°C for 2.75 hours. Digested riboprobe was added to the hybridization buffer and incubated at 42°C for 16 hours. Following two washes with 2 × SSC–0.1% SDS (5 minutes each) and two washes with 0.1 × SSC–0.1% SDS (15 minutes each) RNA was detected using the BrightStar BioDetect kit and exposed to autoradiography film for approximately 16 hours.

To detect SINV genomic and subgenomic RNA species, 5 μg of the same RNA isolated from infected Aag2 cells and mosquitoes was separated on a 1.25% agarose gel containing 0.6 M formaldehyde. The RNA was transferred to a positively-charged Brightstar nylon membrane (Ambion, Inc.) and cross-linked using ultraviolet light. For genomic RNA detection, methods similar to those used for siRNA detection were employed except that all hybridization and wash steps were carried out at 68°C. A biotinylated riboprobe corresponding to SINV genome (11,148–11,320 nt) was generated to detect all three dsSIN viral RNA species.

### Per os mosquito infection

Aliquots of TE/3'2J, TE/3'2J/GFP, and TE/3'2J/B2 virus stocks with pre-determined titers were diluted to 10^7 ^PFU/ml in MEM containing 3% FBS plus NEAA, L-glutamine, and antibiotics. Virus was mixed with warmed defibrinated sheep's blood (Colorado Serum Co., Boulder, CO) and 10 mM adenosine triphosphate (ATP) (45:45:10 v/v) and placed into the central chamber of a water-jacketed glass feeding apparatus using stretched Parafilm (Pechiney Plastic Packaging Inc., Neenah, WI) as an artificial membrane. Mosquitoes that had eclosed five to seven days earlier were allowed to feed for approximately 45 minutes before feeders were removed. Sugar was removed two days prior and water six hours prior to bloodfeeding. Bloodmeal samples were taken post-bloodfeed for virus titer determination. Mosquitoes were cold-anesthetized and engorged females were separated and kept at normal rearing conditions until analysis. All mosquitoes were provided sugar and water *ad libitum*. At four and seven days post oral infectious bloodmeal, 48 individual mosquitoes per virus group were randomly selected. Midguts were dissected from each mosquito and kept in individual tubes. The remaining carcass was placed in a separate tube and paired tubes for each mosquito were kept at -80°C until processing. Individual mosquito tissues were triturated and sterile-filtered. Infectious virus titers were determined by plaque titration as previously described [6].

### Mosquito mortality

For oral infection, five to seven day old female *Ae. aegypti *HWE mosquitoes were given a bloodmeal containing 1 × 10^7 ^PFU/ml of virus or mock-infected cell culture supernatant. One hundred fully engorged mosquitoes were randomly selected and kept at optimal rearing conditions for 21 days. Dead mosquitoes were counted daily for the duration of the experiment.

For intrathoracic injection, mosquitoes were injected with virus or mock-infected culture supernatant using the Nanoject II. Sixty-nine nanoliters of virus (1 × 10^7 ^PFU/ml) or mock supernatant were injected into individual adult female mosquitoes that were cold-anesthetized. Injected mosquitoes were kept at optimal rearing conditions and dead mosquitoes were counted daily for the duration of the experiment. To determine an *Ae. aegypti *50% lethal dose (LD_50_) for TE/3'2J/B2 virus, groups of 50 mosquitoes were injected with 69 nl of virus diluent beginning with a stock virus titer of 1 × 10^7 ^PFU/ml and ending with 1 × 10^2 ^PFU/ml. Injected mosquitoes were maintained and counted daily as previously described [6].

## Abbreviations

SINV: Sindbis virus; VSR: Viral Suppressor of RNA silencing; SGP: subgenomic promoter; PFU: Plaque forming unit; RNAi: RNA interference; dpi: days post-infection

## Competing interests

The authors declare that they have no competing interests.

## Authors' contributions

CMC assisted in the design of the study and wrote the majority of the manuscript. CMC, JCS, and ATP performed the experiments. BJG assisted in the design of the study and helped write the manuscript. KEO assisted in the design of the study, acquired funding for the project, and provided critical analysis of the manuscript.
